# Dutch GPs’ perspectives on addressing obesity: a qualitative study

**DOI:** 10.3399/BJGPO.2023.0112

**Published:** 2024-02-07

**Authors:** Willemijn J van den Hout, Marieke A Adriaanse, Louise M Den Beer Poortugael, Dennis O Mook-Kanamori, Mattijs E Numans, Petra G van Peet

**Affiliations:** 1 Department of Public Health and Primary Care, Leiden University Medical Center, Leiden, The Netherlands; 2 Department of Health, Medical and Neuropsychology, Leiden University, Leiden, The Netherlands; 3 Department of Clinical Epidemiology, Leiden University Medical Center, Leiden, The Netherlands

**Keywords:** overweight, obesity, qualitative research, general practitioners, general practice, primary health care

## Abstract

**Background:**

Early diagnosis and treatment of obesity in primary care may help to tackle the obesity pandemic. Nonetheless, GPs frequently fail to address obesity and demonstrate limited adherence to guidelines.

**Aim:**

To explore Dutch GPs’ perspectives on addressing obesity regarding the following three target behaviours: discussing weight; diagnosing; and referring patients with obesity.

**Design & setting:**

A qualitative focus group study with Dutch GPs.

**Method:**

Six focus groups were conducted with a purposive sample of 21 GPs. Thematic analysis was performed using deductive coding, according to the Theoretical Domains Framework (TDF).

**Results:**

For discussing weight, the main barriers identified were a presented complaint unrelated to obesity (environmental context and resources), concerns about a negative response from the patient (beliefs about consequences), and worries about obesity being a sensitive subject to discuss (emotions). A long-term trustworthy relationship (social influences) facilitated discussing weight. For diagnosing patients with obesity, the main barriers were related to resources; for example, lack of (appropriate) measuring equipment and time (environmental context and resources). For referring patients with obesity, the main barriers were no referral options nearby (environmental context and resources*),* and doubts about the positive effects of the referral on weight change (beliefs about consequences).

**Conclusion:**

Different barriers for discussing weight, diagnosing, and referring patients with obesity were identified, underscoring the importance for tailored interventions to these specific behaviours. Improving knowledge and skills of GPs seems insufficient as this study showed that particular attention should be paid to establishing long-term relationships, addressing GPs' beliefs about consequences, and creating a supportive environment with sufficient time and resources.

## How this fits in

GPs frequently fail to address obesity and demonstrate limited adherence to guidelines. This qualitative study aimed to explore Dutch GPs’ perspectives on addressing obesity regarding discussing weight, diagnosing, and referring patients with obesity. Investment in long-term trustworthy doctor–patient relationships (discussing weight), optimising resources and time management in the consultation room (diagnosing patients with obesity), and improving accessible referral options and beliefs about outcome expectancies (referring patients with obesity)*,* might facilitate addressing obesity in primary care. Future intervention management for addressing obesity should be tailored to each different behaviour for change (discussing, diagnosing, and referring) instead of addressing obesity in general.

## Introduction

The prevalence of patients with obesity is increasing worldwide.^1,2^ In the Netherlands, currently almost half of the population is overweight or obese.^2^ Patients with obesity visit their GP more often than those of a healthy weight,^3,4^ and have an increased risk of morbidity and mortality.^5,6^ This is not only hazardous for patients, but also a burden for primary care, and by extension for the entire healthcare system.^7^ In primary care, it causes a higher workload for the GP and more prescribed medication in this population.^8^ Early identification and explicit diagnosis and targeted treatment approaches for obesity in primary care may help to counteract these negative effects.

Nonetheless, in daily practice GPs often fail to address obesity and experience difficulties adhering to the practice guidelines.^9,10^ This is unfortunate, since GPs are in a crucial position in the healthcare system to signal, diagnose, and treat patients with obesity. The national guideline for obesity of the Dutch College of General Practitioners (NHG) describes when diagnostics, treatment, and referral are indicated.^11^ Understanding why there is limited adherence to these guidelines regarding obesity care requires insight into the determinants of the GP's behaviour regarding addressing obesity.

A successful approach to addressing obesity in primary care requires the GP to perform different behaviours; for example, discussing weight, diagnosing, and referring patients with obesity for treatment of their obesity. Different barriers may exist for each of these behaviours. In order to understand determinants of behaviour and to facilitate behaviour change, there is a need for the behaviour for change to be specified and clearly selected.^12^ Previous research on perspectives of GPs for addressing obesity and adherence to obesity guidelines did not specify the assessed behaviours upfront.^13–15^ In the present study, we address this limitation by focusing on three specific behaviours separately using the Theoretical Domains Framework (TDF) as a framework, specifically designed to understand determinants of healthcare professional behaviour.

The TDF consists of 14 theory-based domains that represent varying determinants for behaviour change; for example, knowledge, environmental context and resources, social influences, and beliefs about consequences.^16,17^ This evidence-based approach was developed to assess implementation problems and health professional behaviours as a basis for intervention development.^17^ Each domain of the TDF relates to a component in the overarching Capability, Opportunity, Motivation, and Behaviour (COM-B) model. This model identifies the following three key factors that need to be present for any behaviour to occur: capability; opportunity; and motivation.

To our knowledge only one study used the TDF to explore barriers and facilitators of healthcare professionals in addressing obesity.^18^ However, this study focused only on discussing weight, whereas effective management of obesity in primary care also requires essential behaviours such as diagnosing and referring patients with obesity. With the present study, we thus aimed to extend on these findings by applying the TDF to explore the barriers and facilitators of GPs for three specific target behaviours that are crucial to adhere to the guidelines: discussing weight; diagnosing patients with obesity; and referring patients with obesity.

## Method

### Design and setting

This study is a qualitative study using the outcome of tightly guided focus group discussions. Focus groups were chosen as it has been shown that focus groups allow for participant interaction and group dynamics, which may provide a broader range or scope of perspectives and information.^19^ Focus groups were organised with GPs working in primary care in the Netherlands.

### Participant selection and recruitment

We used purposive sampling to recruit a heterogenous sample of GPs in terms of age, sex, working experience, GP practice setting, and patient populations. We recruited GPs from the extramural Leiden University Medical Centre (LUMC) academic network (ELAN), an online platform for GPs (HAweb), a local network of locums, and from the personal network of the researchers. Potential participants received written information regarding study purposes and provided written informed consent before participation. Focus groups were organised with three to five participants, and new groups were added until data saturation was reached (that is, until no new themes were brought forward).

### Data collection

In each focus group the following three specific target behaviours were discussed: discussing weight; diagnosing; and referring patients with obesity. Discussing weight referred to raising the topic of weight during consultation. Diagnosing patients with obesity referred to measuring height, weight, and preferably also waist circumference, followed by structured recording the measurements in the electronic health record (EHR). Referring patients with obesity for treatment included various options; for example, a dietician, a lifestyle coach, a combined lifestyle intervention (CLI; combining healthy diet, physical activity, sleep and stress management), the general practice nurse, and bariatric surgery. A semi-structured topic guide for each target behaviour was developed based on the 14 domains of the TDF (Supplementary Table S1). For each target behaviour, participants were asked questions related to all 14 domains of the TDF to gain insight into the barriers and facilitators.

Before the start of each target behaviour, we showed participants one of the three vignettes of an encounter with a specific patient with obesity, as an example to prompt GPs with a variety of real-life practice situations. The vignettes included the following:

- for discussing weight, a patient with obesity with a reason of encounter unrelated to obesity;
- for diagnosing patients with obesity, a patient with obesity asking for help to lose weight;
- and for referring patients with obesity, a patient with obesity with cardiovascular risk factors (Supplementary File S1).

The focus groups lasted 2 hours and were all moderated by an experienced moderator (PP) assisted by two observers (WH, LB) who made fieldnotes. The first and second focus groups took place in the LUMC. The next four focus groups were conducted online as COVID-19 restrictions hindered coming together in person. Data collection took place between September 2021 and February 2022. The focus groups were audiorecorded, and transcribed verbatim by two researchers (WH, LB).

### Data analysis

The transcripts were analysed using a thematic analysis approach using Atlas ti (version 22). The 14 theoretical domains of the refined TDF were used for deductive coding.^16,20^ Barriers and facilitators were identified within each domain. If content did not fit in one of the pre-specified TDF domains, an additional (inductive) code was added. To structure the result section of the report, the COM-B system was used.^12^ Two researchers (WH, LB) independently coded the focus group discussion to increase reliability. To resolve any inconsistencies and coding problems and to refine generated themes, the research team (including a behavioural scientist; MA) frequently discussed allocation of the codes and themes to TDF domains until agreement was reached.

## Results

### Sample characteristics

**Figure 1.**
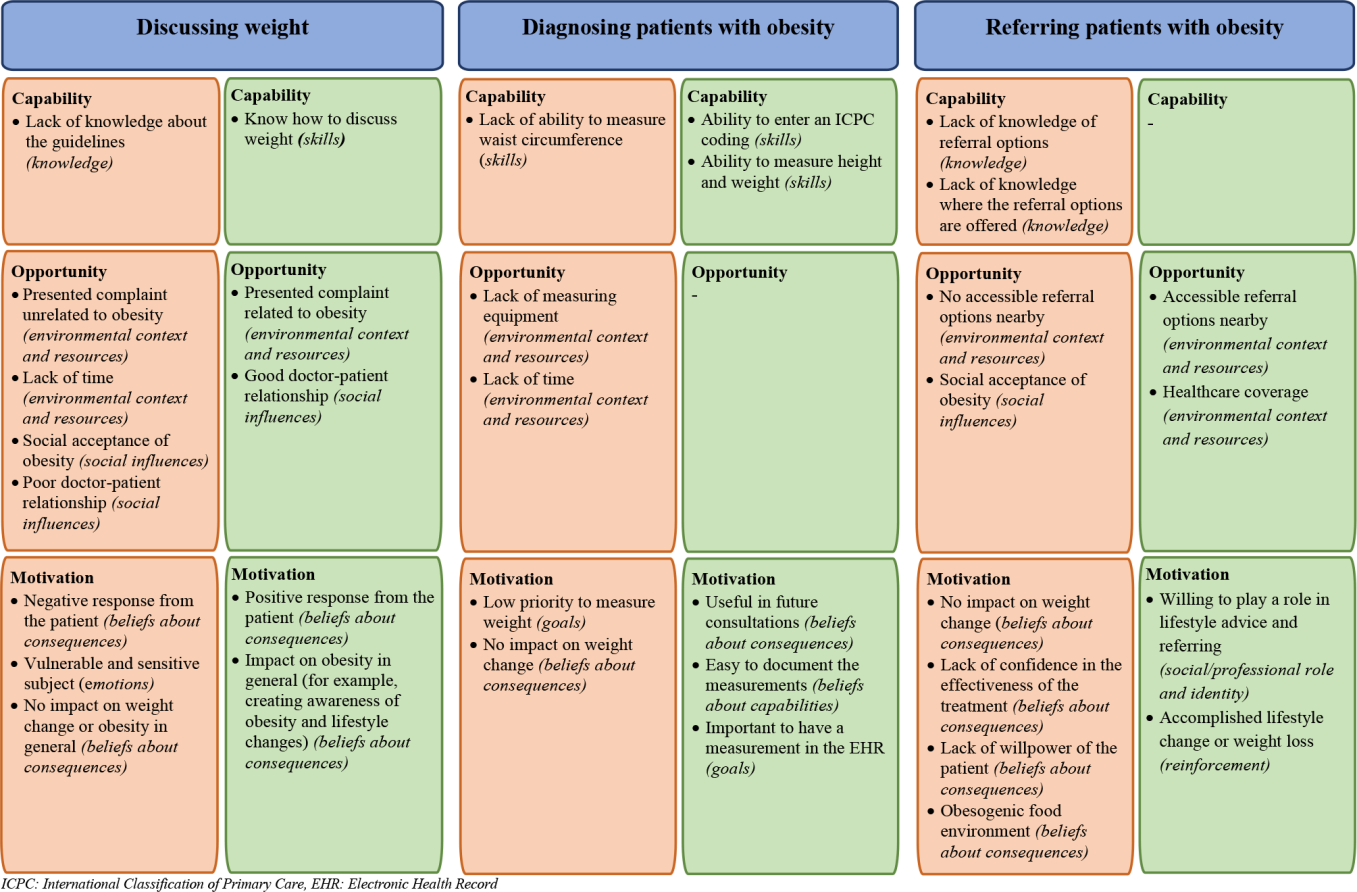
Main barriers and facilitators regarding the three target behaviours structured into the Theoretical Domains Framework (TDF) and Capability, Opportunity, Motivation,and Behaviour (COM-B) model. EHR = electronic health record. ICPC = International Classification of Primary Care.

We reached data saturation after six focus groups with three to five GPs (*n* = 21). Table 1 presents the characteristics of the study population. The participants had a mean age of 49 years (range 33–66 years) and the majority were women (76.2%). For each target behaviour, the main barriers and facilitators structured into the three COM-B components with the related TDF domain in brackets are described below. Figure 1 summarises these barriers and facilitators. Supplementary Table S2 summarises all reported barriers and facilitators for each domain of the TDF.

**Table 1.** Sample characteristics reported by the participants (*n* = 21)

Characteristic	*n*
Age, years	
30–39	6
40–49	6
50–59	6
60–69	3
Sex	
Female	16
Male	5
Experience as GP, years	
0–9	8
10–19	5
20–29	6
30–39	2
Type of employment	
Practice owner	10
Salaried service	2
Locum	9
Practice location	
Urban	12
(Semi) rural	8
Both	1
Type of practice	
Solo practice	8
Duo practice	5
Group practice	5
Mixed	2
Unknown	1
Number of patients in practice	
≤3000 patients	9
>3000 patients	9
Unknown	3
Type of patient population	
Average population (reflection of the Dutch population)	10
Other	11
Specific areas of interest	
GP trainer	7
Obesity	1
Lifestyle coach	1
Other	6
None	6

### Discussing weight

#### Capability

In the domain of capability, knowledge was the only barrier mentioned related to capability. Several GPs indicated that they had insufficient knowledge regarding guidelines for addressing obesity. Several facilitators were mentioned related to capability. Some GPs mentioned feeling competent in discussing weight. They emphasised they possessed the skills to discuss weight by fact-focused communication and by using the correct vocabulary (skills). Another facilitator was a documented body mass index (BMI) measurement in the EHR as some participants indicated this functioned as a reminder for discussing weight at follow-up (memory, attention, and decision processes).

#### Opportunity

An important barrier for discussing weight mentioned in all focus groups, was the difficulty to discuss weight when the presented complaint was unrelated to obesity (environmental context and resources). When complaints were related to obesity (for example, joint complaints, cardiovascular risk factors, infertility, or diabetes) a conversation about weight was said to be easier to start:

*‘If the complaint they come up with is unrelated to obesity, I find it to be almost inappropriate to start a conversation about obesity (…) I really must have a clear relationship with obesity, for example, cardiometabolic diseases, fatigue or anything else I can comment on …*’ (GP 16)

Within this domain (environmental context and resources), lack of time was mentioned as a barrier to discuss weight, particularly when the GP was inexperienced, was unfamiliar with the patient, or worked as a locum. Social influences were mentioned both as an important barrier and facilitator for discussing weight. Specifically, the absence of a pre-existing good doctor–patient relationship was mentioned as a barrier especially by locums. On the other hand, having a good doctor–patient relationship facilitated discussing weight. This good relationship could arise from a positive atmosphere during consultation, from building a relationship of trust, from experience or from being familiar with the patient.

#### Motivation

Beliefs about consequences was another important barrier for discussing weight and was mentioned in all focus groups. GPs were hesitant to discuss weight owing to fear of negative responses, which might harm their doctor–patient relationship. However, other GPs mentioned never having negative responses from patients, which facilitated discussing weight:

*'People never respond, “mind your own business”, but I must say I know these people for a long time (…) they know my intentions.*’ (GP 10)

Albeit less frequently discussed, GPs were unconvinced about their influence on weight change or the problem of obesity in general by discussing weight (beliefs about consequences). As a facilitator, a few GPs pointed out that they felt they could influence obesity by creating awareness, promoting lifestyle changes, or preventing comorbidities. Anticipated emotions were also a mentioned barrier for discussing weight. GP*s* expressed feeling reluctant to discuss weight, as they considered it a sensitive subject (emotions):

*' … people may be embarrassed about it or find it a sensitive subject, which makes it difficult for me to bring it up.’* (GP 18)

Finally, a new theme that did not fit the existing TDF framework emerged and was therefore inductively added as a new theme in our analysis: characteristics of the patient. Characteristics of the patient (for example, age) were said to function either as a barrier or a facilitator for discussing weight. Almost all GPs had examples of patient characteristics (age, sex, BMI, motivation, comorbidities and socioeconomic status of the patient) that they felt made it easier or more difficult to discuss weight. Some characteristics were mentioned as a facilitator by some but as a barrier by others. GPs who mentioned a specific characteristic explained why it was easier or more difficult to discuss weight with a patient with this characteristic. For example:

*'I am more reluctant with men because they do not like me nagging*.’ (GP 2)

*' … the younger the patient is, the more likely you are to achieve health benefits …’* (GP 8)

*'Healthy food is expensive, for example if a patient has financial problems, it is not that easy to eat healthily. For this reason, I will not discuss weight.’* (GP 2)

### Diagnosing patients with obesity

#### Capability

Domains related to capability were not frequently mentioned for diagnosing patients with obesity. As a barrier, some did indicate a lack of skill in measuring waist circumference. As facilitator, GPs knew how to enter an International Classification of Primary Care (ICPC)-coding and document the measurements in the EHR (skills).

#### Opportunity

Almost all barriers mentioned in diagnosing patients with obesity were in the domain of environmental context and resources. Specifically, lack of (appropriate) materials in consultation rooms (for example, scales and measuring tape) was mentioned as a barrier, especially by some locum GPs without their own consultation room. Lack of time was also sometimes mentioned.

#### Motivation

The most important facilitator for diagnosing patients with obesity, mentioned in all focus groups was that GPs measure and document obesity since it helps themselves in future consultations. For example, when discussing weight at follow-up, assessing cardiovascular disease at follow-up, writing a referral, prescribing medication, it was useful to have an adequate weight in the EHR. Another reason to document obesity was to facilitate easier collaboration with colleagues (beliefs about consequences):

*'It is good to document weight because it also affects other conditions. I sometimes see patients of a colleague and have to interpret laboratory results. To be able to do this, you need to know if someone is overweight, just as when prescribing. So, it is good to document.’* (GP 21)

*' ... if I document obesity then I can later bring up the subject more easily.’* (GP 18)

Another barrier mentioned by GPs was that documenting obesity was not their priority in daily practice, but as a facilitator they considered it was important to document it in the EHR (goals).

### Referring patients with obesity

#### Capability

For capability, mainly topics belonging to the domain of knowledge were discussed. As a barrier, GPs mentioned a lack of knowledge about referral options, or where the referral options are offered in their municipality, particularly for lifestyle coaches and CLIs. Some GPs had also insufficient knowledge about criteria for certain referral options. Most GPs were able to find a dietician (knowledge).

#### Opportunity

The first most important barrier mentioned for referring patients with obesity involved the domain environmental context and resources. Specifically, lack of availability of accessible referral options nearby was mentioned as a barrier:

*'We do not use the combined lifestyle intervention because there are no healthcare providers who offer this in our city …’* (GP 11)

In contrast, having accessible referral options nearby (for example, through personal contact with the healthcare providers or offered treatment on-site) was mentioned as a facilitator by some GPs. Also, healthcare coverage for treatment of obesity was mentioned as a facilitator (environmental context and resources). Lastly, a less frequently mentioned barrier was that GPs failed to refer since obesity has become socially accepted (social influences).

#### Motivation

The other most important barrier for referring patients with obesity concerned beliefs about consequences. In all focus groups, GPs doubted the impact the referral could have on obesity or weight change. This doubt had several reasons: first, GPs mentioned that they had little confidence in the healthcare providers they could refer to, especially dieticians. They mentioned disappointing results and patient dropouts owing to lack of motivation:

*' I have not always been enthusiastic about the dietician in our village (…) although they are not doing too bad, it does not always yield a lot in terms of losing weight.’* (GP 6)

*'… that dietician from whom I received the third letter from, stating that someone dropped out. At that moment I think I should not do this anymore.’* (GP 11)

Second, confidence in the effectiveness of the CLI differed between GPs. Some were convinced of its effects while others mentioned a lack of evidence, long-term results, and lack of willpower of the patient:

*'… I am glad I have got the option of a combined lifestyle intervention, as this allows me to refer the patient, but that does not mean I am sure about its effects yet.’* (GP 8)

Third, some GPs were hesitant to refer for bariatric surgery, as they had encountered the disadvantages after surgery, and they doubted the long-term effectiveness. Lastly, GPs doubted the impact their referral could have owing to the obesogenic food environment with unhealthy cheap foods being omnipresent (beliefs about consequences). Within this domain (beliefs about consequences) a facilitator was that GPs found it easier to refer patients with obesity for reasons such as preventing comorbidities, achieving health benefits, or maintaining a stable weight.

GPs were in doubt about their professional role in obesity. They were all sure they should create awareness of obesity and should discuss weight and the problems associated with it, but uncertain about their exact role in the follow-up. Some GPs were eager to treat patients with obesity themselves, while other GPs felt they would rather refer. Many GPs also acknowledged a role for the community and government; for example, tax on sugar and regulations regarding obesity at school (social or professional role and identity):

*'… our society is so sickening, when you walk into a supermarket, you first pass the cookies, chocolate, and sweet drinks. It is not something for just the GP to address, it is also a societal task.’* (GP 14)

Finally, a new theme for referring patients with obesity was once again the characteristics of the patient (inductively added). For this target behaviour, this was mainly mentioned as a barrier. In all focus groups, GPs found it difficult to refer their patient if they noticed a lack of motivation during consultation. In addition to this barrier, a low socioeconomic status (for example, patient is unable to afford the treatment or healthy food) was also mentioned as a barrier.

## Discussion

### Summary

This focus group study explored GPs’ barriers and facilitators in discussing weight, diagnosing, and referring patients with obesity related to the TDF. For discussing weight, the main barriers identified were related to environmental context and resources, beliefs about consequences, and emotions. GPs failed to discuss weight when the presenting complaint was unrelated to obesity, when they were concerned about a negative response from the patient, and when they worried about obesity being a sensitive subject. For diagnosing patients with obesity, the most important barrier was related to environmental context and resources; for example, lack of (appropriate) measuring equipment and time. For referring patients with obesity, the main barriers were related to beliefs about consequences*,* knowledge, and environmental context and resources. GPs doubted about the positive effects of the referral on weight change, had insufficient knowledge of referral options, and had a lack of accessible referral options nearby. In summary, different barriers and facilitators existed for discussing weight, diagnosing, and referring patients with obesity, which has indicated the necessity to tailor future interventions to each specific behaviour. Moreover, our findings have suggested that limited knowledge and skills are not major barriers to any of the behaviours. Interventions should rather pay particular attention to barriers such as addressing beliefs about consequences and creating a supportive environment with sufficient time and resources.

### Strengths and limitations

Strengths of this study included the systematic way in which the problem was approached and defined. First, in line with step two (select the target behaviour) and step three (specify the target behaviour) of the behaviour change wheel,^12^ three specific target behaviours were specified and addressed in the focus groups. Second, we used the TDF, which is the most widely used, integrated theoretical framework for understanding healthcare professional behaviour, and which allows for identifying a broad range of facilitators and barriers in a structured manner. Results revealed that for the specific target behaviours, the barriers and facilitators were on different domains within the TDF, which implied that different behaviour change techniques will be required to support GPs for the different behaviours. Some limitations should be taken into account. First, focus groups could yield more socially acceptable answers. Second, the participating GPs might have had a special interest in obesity and may have been more motivated to optimise the care for patients with obesity. However, it is to be noted that participants were asked about their special interests in general practice and only two GPs expressed having a special interest in obesity care or lifestyle medicine (Table 1). Lastly, the risk of bias resulting from the use of the vignettes in the focus groups must be mentioned. We aimed to start the broad discussions about each target behaviour with a realistic and representative vignette to enliven their memories of real-life practice situations, but the perspectives of the GPs may have been influenced by the examples we used, which were different for the three behaviours.

### Comparison with existing literature

For discussing weight, this study confirmed the difficulty in discussing weight when the presented complaint is unrelated to obesity.^18,21–24^ Additionally, in our study many GPs agreed that their knowledge of obesity, its risks, and the skill on how to start a conversation were sufficient, this was in contrast with two previous studies that mentioned the uncertainties on the level of knowledge about obesity being a medical condition.^18,25^ Concerning diagnosing patients with obesity, it has been shown that GPs often fail to document obesity in the EHR,^26,27^ especially for patients with obesity who are younger and without comorbidities.^27,28^ To our knowledge, the reasons behind this under-recording have not been investigated before. Regarding referring patients with obesity, GPs were in doubt about the effectiveness of the referrals on weight changes. This is underpinned by studies showing only modest weight reduction of dietary interventions.^29–31^ Also, the long-term effectiveness of the CLI is still uncertain and has not been proven yet.^32–36^ In addition, GPs admitted their limited knowledge of CLIs, as confirmed by van der Heiden *et al*.^37^

Some challenges were experienced when mapping the data onto the TDF. Therefore, we added a new code in our analysis: characteristics of the patient (for example, age, sex, socioeconomic status). Almost all GPs had examples of a type of patient they felt easier or more difficult to discuss weight with. This is in line with a study showing differences in addressing obesity in patients with specific characteristics in clinical practice.^38^ They found an association between addressing obesity and the female sex, socioeconomic deprivation, non-White ethnic group, comorbidities, and the heaviest BMI group. These findings and our findings indicated that addressing obesity is a complex problem and requires a patient-centred approach, which involves personalised care for each specific patient characteristic.

### Implications for practice

To address these different barriers and facilitators within each target behaviour, it is important to acknowledge the need for tailored interventions management for each specific behaviour.

For discussing weight, establishing strategies for discussing sensitive topics and training in communication techniques might facilitate the GP to discuss weight even when the complaints are unrelated to obesity or when the GP is worried about a negative response from the patient. Also, long-term trustworthy doctor–patient relationships and patient–provider continuity are important to this end. This is a challenge since the number of locum GPs has been increasing over the past years in the Netherlands; this aspect needs specific attention in primary care.^39–41^

For diagnosing patients with obesity, it is important to acknowledge the lack of environmental resources and time during consultation. Routinely measuring and weighing patients with obesity and recording the results by the practice nurse before entering the consultation room might be helpful. Also, supplying scales and measuring tapes in each consultation room should be considered.

For referring patients with obesity, awareness of available referral options, easy access to nearby options, and confidence in the expected outcomes are essential. Studies showed that awareness and knowledge among GPs regarding content and effectiveness of healthcare innovations, such as CLIs, are crucial for developing a positive attitude towards these innovations.^37,42–44^ Therefore, providing education and involvement of the GP could contribute to increased referrals to CLIs. A positive development is that healthcare insurances have started to reimburse CLIs in January 2019 in the Netherlands.^45^ GPs in our study agreed that healthcare coverage for such treatments facilitates referral.

Finally, since GPs mentioned that they felt the problem of addressing obesity goes beyond the scope of the GP’s profession, it is of utmost importance that obesity is also addressed by politicians at a societal level.^13,46,47^

In conclusion, based on our results, investment in long-term trustworthy doctor–patient relationships (discussing weight), optimising resources and time management in the consultation room (diagnosing patients with obesity), improving accessible referral options, and addressing beliefs about outcome expectancies (referring patient with obesity) are likely to facilitate addressing obesity in primary care. Future intervention management should be tailored to each different behaviour for change (discussing weight, diagnosing, and referring patients with obesity) rather than on addressing obesity in general. Additionally, since most barriers and facilitators concerned beliefs about consequences and environmental context and resources, these should be taken into account when developing future interventions. Adjusting guidelines and improving knowledge among GPs is part of the solution, but by itself insufficient to address obesity in primary care.

## Data

All data relevant to the study are included in the article or uploaded as online supplemental information.
